# Classification of prestrike types in vacuum interrupter eroded by inrush current based on DC dynamic gap measurement method

**DOI:** 10.1371/journal.pone.0328461

**Published:** 2025-07-15

**Authors:** Pu Chen, Yun Geng, Jing Yan, Hannan Shan, Yan Wang, Zhiyuan Liu, Hanyan Xiao, Tianxin Zhuang

**Affiliations:** 1 State Key Laboratory of Electrical Insulation and Power Equipment, Xi’an Jiaotong University, Xi’an, Shaanxi Province, People’s Republic of China; 2 School of Electrical Engineering, Xi’an University of Technology, Xi’an, Shaanxi Province, People’s Republic of China; 3 State Grid Jiangsu Electric Power Co. Ltd. Research Institute, Nanjing, Jiangsu Province, People’s Republic of China; University of Oxford, UNITED KINGDOM OF GREAT BRITAIN AND NORTHERN IRELAND

## Abstract

The characteristics and types of prestrike are critical factors affecting the performance of vacuum circuit breakers (VCBs) in controlled switching. However, there is a lack of studies on the prestrike characteristics and types in vacuum interrupter (VI) after inrush current erosion. This paper investigates the prestrike characteristics and types in VI after inrush current erosion, using a novel method for measuring field emission current under DC voltage. Through experiments, the prestrike characteristics after inrush current erosion were obtained, including prestrike dispersion and prestrike gap. Furthermore, based on the prestrike gap and field emission current, the prestrike types observed in the experiments were classified into three categories: field emission induced prestrike (FEPS), particle-induced prestrike (PPS) and field emission-particle induced prestrike (FE-PPS). The average prestrike gap for these three types were 1.525 mm, 3.809 mm, and 2.887 mm, respectively. PPS showed no significant field emission current, while the field emission current at prestrike moment for FE-PPS was approximately 48.89% of that observed in FEPS. These findings have important implications for the development of controlled switching technology in VCB.

## Introduction

With the rapid development of technology, electric power has become an indispensable driving force for economic and industrial development [1]. In parallel, expectations for power quality and power system stability have intensified alongside technological proliferation [2]. In power systems, the widespread presence of capacitive and inductive loads leads to substantial reactive power generation during operation. This reactive power increases system losses and adversely affects stability. To address these challenges, capacitor banks switching is widely employed for reactive power compensation [3]. Vacuum circuit breakers (VCBs) offer notable advantages for capacitor banks switching due to their high operational reliability, environmentally friendly design, and self-contained vacuum interrupter (VI) [4].

However, comparative studies indicate that VCBs are significantly more prone to restrike phenomena than SF₆ circuit breakers when switching capacitor banks at similar voltage levels, which greatly limits their applicability in such scenarios [5,6]. Restrikes can result in severe overvoltages. These transient overvoltages pose serious risks to equipment integrity, threatening overall grid stability and operational safety [7]. Contact surface erosion caused by inrush current during the prestrike process has been identified as a major contributor to restrike occurrence [8,9]. The inrush current can erode the contact surface, creating localized fusion welds. Upon contacts separation, these welds rupture, damaging the surface and generating defects such as micro-protrusions, pits, and metallic particles [10,11].

To mitigate contact erosion from inrush current, controlled switching has been introduced for capacitor banks operations [12]. The prestrike characteristics and the dispersion of prestrike gap are key factors in developing controlled switching [13]. Current research on prestrike characteristics only considers VI unaffected by inrush current erosion [14,15]. Inrush current erosion induces permanent defects on VI contact surfaces. These defects alter the prestrike characteristics of VI. Studying the prestrike characteristics of VI subjected to inrush current erosion is essential for advancing controlled switching technology. However, there is currently a lack of research on the prestrike characteristics of VI after inrush current erosion.

On the other hand, different types of prestrike lead to different prestrike gaps, which affects prestrike dispersion. Accurately identifying the prestrike type is important for improving the performance of controlled switching and VCB insulation [16]. Classifying prestrike types requires simultaneous measurement of the voltage of VI, field emission current, and contact travel curves during contact closing. However, conventional measurement methods involve adjusting the contact gap to a fixed distance and then applying AC voltage to measure the field emission current [17,18]. This approach cannot simultaneously measure the field emission current and the contact travel curve. Consequently, current research has only explored vacuum breakdown types at fixed contact gap. Studies focusing on prestrike types during actual contact closing remain unexplored [19,20].

**Fig 1:**
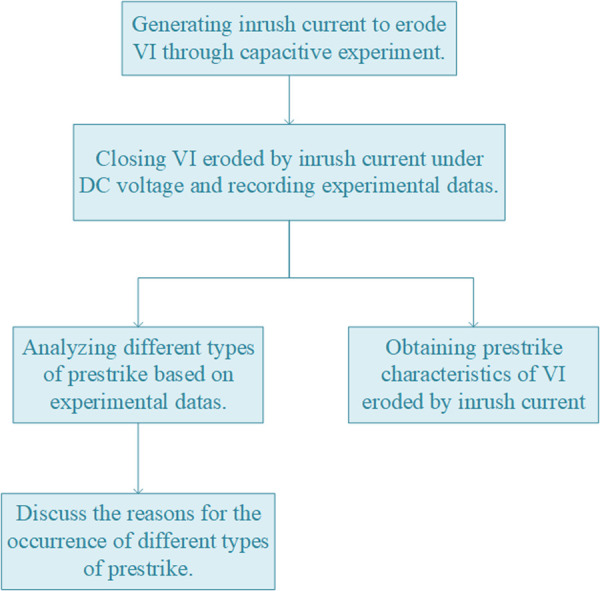
The research flow chart for this study.

Recently, a new method for measuring field emission current under DC voltage has enabled the classification of prestrike types [21]. Based on this approach, this paper simultaneously investigates both prestrike characteristics and prestrike types in VI eroded by inrush current. Fig 1 illustrates the research flow of this study. Initially, the VI is eroded by generating inrush current through capacitive switching experiment. After completing the capacitive switching experiments, DC prestrike characteristic experiments are performed on the eroded VI. During contact closing, the voltage of VI, field emission current, and contact travel curves are measured simultaneously. Subsequently, prestrike types are classified and prestrike characteristics of the VI are calculated based on experimental data. Finally, the experimental results are discussed considering the physical mechanisms underlying prestrike types.

### Experimental setup

**Fig 2:**
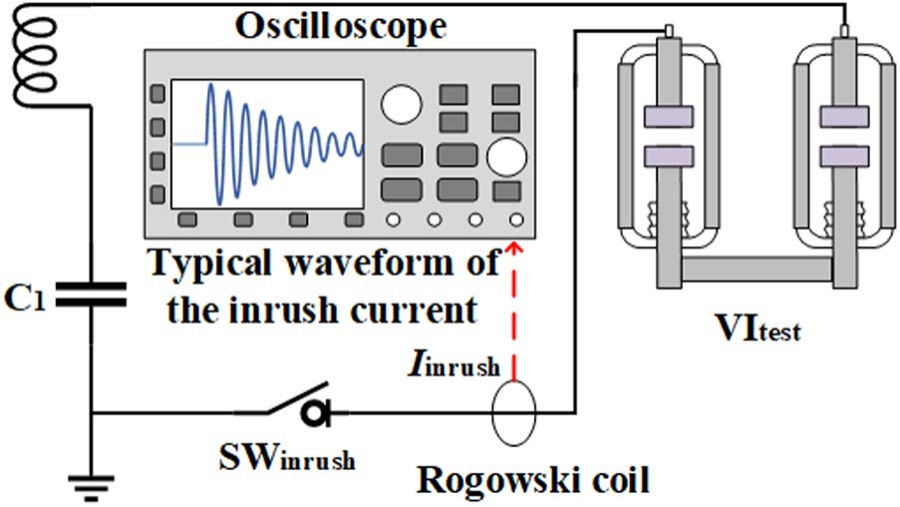
The schematic diagram of the capacitive switching experiment circuit.


The VI applied in controlled switching applications is usually eroded by inrush current. In order to reproduce the real state of VI after being eroded by the inrush current, this paper generates the inrush current to erode the VI through the capacitive switching experiment. Fig 2 shows the schematic diagram of capacitive switching experiment circuit and illustrates a typical inrush current waveform. The capacitive switching experiment circuit mainly consists of circuit breaker SW_inrush_, capacitor bank *C*, inductor *L*, test vacuum interrupter VI_test_ and Rogowski coil. The parameters of the inductor *L* and capacitor *C* in the circuit together determine the frequency *f* of the inrush current, as shown in Equation (1).

$$f=\frac{w}{2\pi }=\frac{1}{2\pi \sqrt{LC}}$$ (1)

The amplitude *I*_*m*_ of the inrush current is determined by charging voltage *U*_*m*_, the capacitor bank *C*, and the inductance *L*, as shown in Equation (2).


$$I_{\text{m}}=U_{\text{m}}wC=U_{\text{m}}\sqrt{\frac{C}{L}}$$ (2)

**Fig 3:**
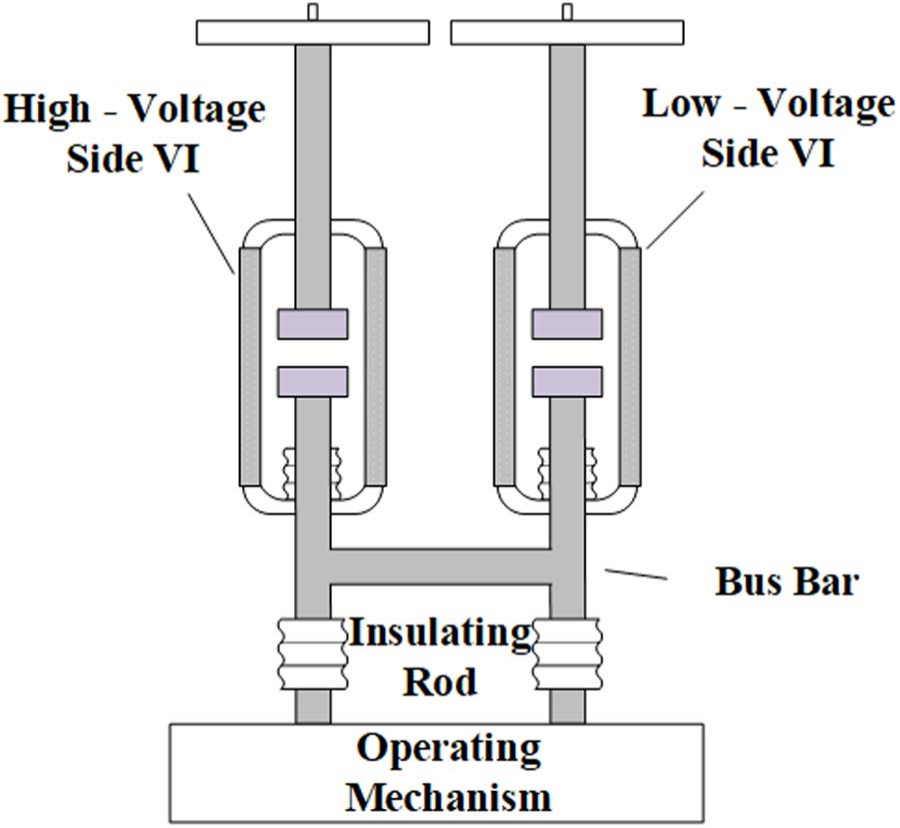
The schematic diagram of the double-break vacuum circuit breaker.

The experiment was carried out on a double-break VCB consisting of two commercial 12 kV VI of the same type connected in series, as shown in Fig 3. Before the experiment, both SW_inrush_ and VI_test_ are kept disconnected and *C*_*1*_ is charged to a fixed voltage. The signal is then triggered to close SW_inrush_ first, followed by closing VI_test_. During the closing process of VI_test_, as the contacts in the VI_test_ continue to approach, the electric field strength between the contacts will continue to rise. When the electric field strength between the contacts exceeds the vacuum gap insulation strength, prestrike will occur. Subsequently, the inrush current passes through the VI and creates a prestrike arc until the contact is fully closed. The inrush current gradually oscillates and decays to zero due to the circuit resistance.

**Fig 4:**
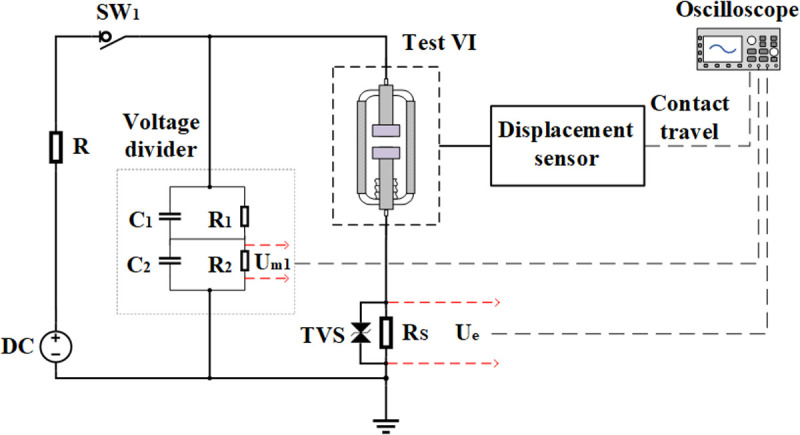
The experimental circuit schematic diagram of DC prestrike characteristics.

Fig 4 shows the experimental circuit schematic diagram of prestrike characteristics under DC voltage, which is mainly composed of a DC voltage source, resistor *R*, voltage divider, test vacuum interrupter VI_test_, displacement sensor and resistor *R*_*s*_ parallel to the TVS. Short one of the VIs in series on the double-break VCB, and connect the other VI to the experimental platform to form a single VI_test_ for the experiment. During the DC prestrike characteristics experiment, the resistor *R*_*s*_ is in series with the VI_test_ to measure the field emission current with the oscilloscope and the resistor is a non-inductive resistor with a resistance of *R*_*s *_= 200 Ω. Resistor *R*_*s*_ is in parallel with a transient voltage suppressor (TVS) to protect the oscilloscope from overvoltage. The breakdown voltage of the TVS is 83.3 V and the reverse standoff voltage is 75 V. The voltage waveform of the VI_test_ can be measured through the voltage divider, and the contact travel curve can be recorded through the displacement sensor at the same time. The displacement sensor is a GEERT linear displacement sensor with a resolution of 0.01 mm and a maximum working speed of 10 m/s.

The current (*i*) measured through R_s_ is divided into three components: field emission current (*i*_*fe*_), displacement current (*i*_*c*_) and leakage current (*i*_*leak*_). The relationship between them is shown in Equation (3):


$$i=\frac{U_{e}}{R_{s}}=i_{fe}+i_{c}+i_{leak}$$ (3)

Where *U*_*e*_ is the voltage of *R*_*s*_ and *R*_*s*_ is the resistance.

The resistance of the insulator is about 10^14^ Ω and applied DC voltage is 30 kV, ensuring that the leakage current remains below 1 µA. Therefore, the leakage current *i*_*leak*_ can be disregard. For displacement current (*i*_*c*_), the literature [21] suggests that the effect of displacement current can be eliminated by applying a low power DC voltage source. The voltage source used in this experiment is a low power DC voltage source, so the displacement current can be neglected. In summary, it can be assumed that the current (*i*) measured through *R*_*s*_ is basically equal to the field emission current (*i*_*fe*_).

**Fig 5:**
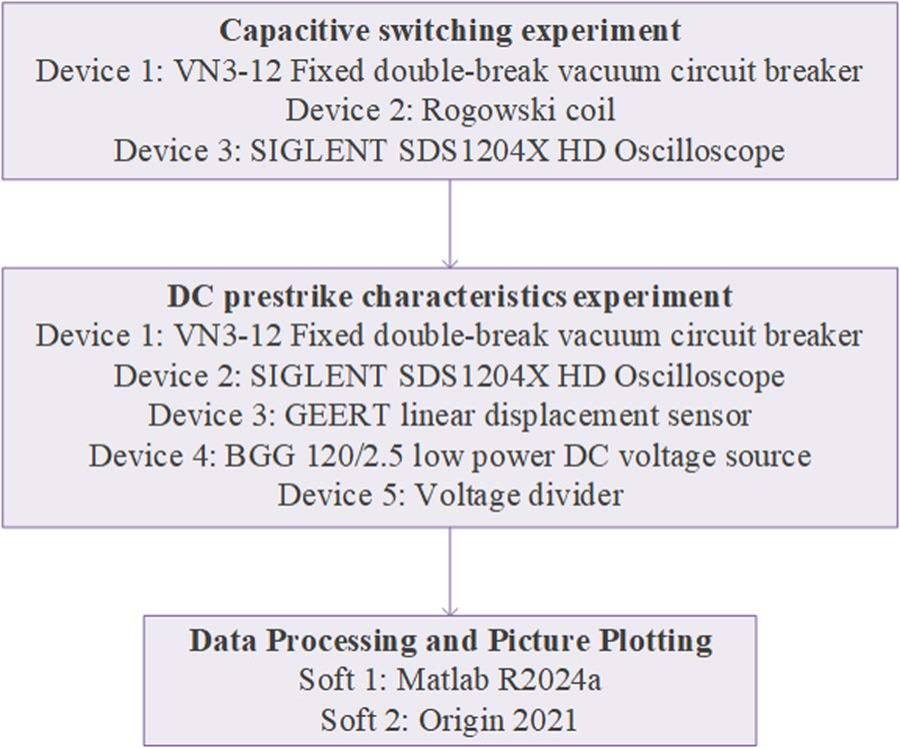
The specific experimental device and software used in this study.

Fig 5 illustrates the specific experimental devices and softwares used in this study. The various settings of the experiment are summarized in Table 1. The VCB tested in this experiment is a double-break VCB with cup-type axial magnetic field (AMF) contact and the material of contact is CuCr30. The experiments were conducted under ambient conditions, specifically at a temperature of 17 ± 2 °C and a relative humidity of 47 ± 3%. For the experiment of capacitor banks switching, the international electrotechnical commission standard recommends test values for circuit breaker with inrush current of 20 kA at 4250 Hz [10]. Therefore, the inrush current amplitude and frequency of the capacitive switching experiments were 20 kA, 4250 Hz, and the experiments were repeated 80 times. After the capacitive switching experiment, the high-voltage side VI (VI_H) and the low-voltage side VI (VI_L) of the double-break VCB were removed. In the DC prestrike characteristics experiment, VI_H and VI_L were each subjected to 200 closing operations under DC voltage. The rated voltage of the VI used in this experiment was 12 kV. For safety considerations, the test voltage was set to 30 kV DC. At this voltage level, the phenomenon of prestrike can be clearly measured by oscilloscope.

**Table 1:** Experimental setup for capacitive switching experiment and DC prestrike characteristics experiment.

VI_test_	VI_H	VI_L
Contact structure	Cup-type AMF contact	Cup-type AMF contact
Contact material	CuCr30	CuCr30
Inrush current	20 kA, 4250 Hz	20 kA, 4250 Hz
Switching test duty	80	80
Closing DC voltage times	200	200
DC Voltage	30 kV	30 kV

### Classification of the prestrike types

**Fig 6:**
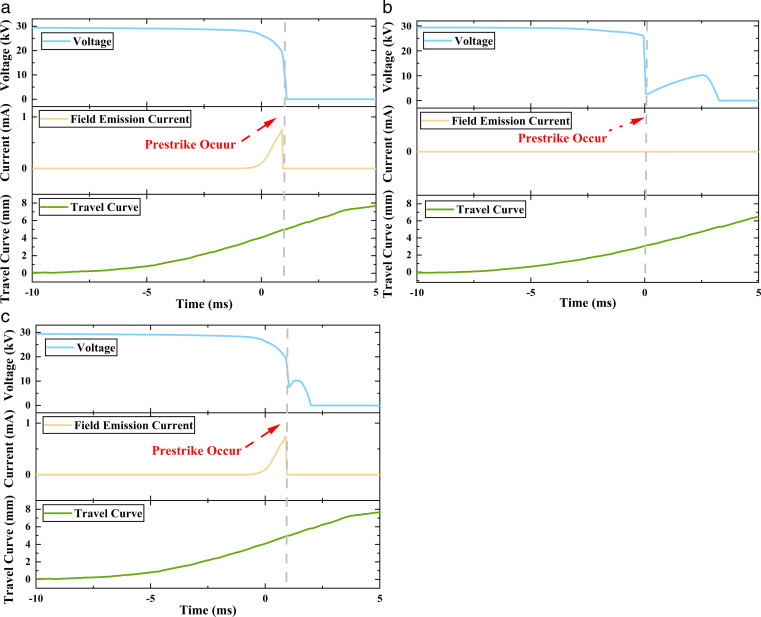
Three different types of prestrike waveform. (a). Prestrike A. (b) Prestrike. B (c). Prestrike C.

After analyzing the field emission current, the voltage of the VI, and the travel curve waveform during the closing process, it was found that there existed three different types of prestrike waveforms (Prestrike A, Prestrike B, and Prestrike C), as shown in Fig 6.

Fig 6(a) presents a typical waveform of Prestrike A. During the closing operation, the prestrike occurs before the contacts are fully closed. Prior to the prestrike, the field emission current increases exponentially, accompanied by a corresponding exponential decrease in VI voltage. At the prestrike moment, both the field emission current and the VI voltage abruptly drop to zero.

Fig 6(b) illustrates the waveform of Prestrike B, which occurs in the presence of particles. The prestrike initiates before the contacts are fully closed, marked by a sudden drop in VI voltage to a residual potential. Following this, the voltage partially recovers owing to insulation recovery. When the electric field strength exceeds the dielectric strength, the voltage will gradually decrease to zero. Notably, no significant increase in field emission current is observed before the prestrike.

Fig 6(c) shows the waveform of Prestrike C, which results from the combined influence of particles and field emission current. Similar to Prestrike B, the voltage of the VI drops abruptly before the contacts are fully closed, followed by a partial recovery and exponential decay. However, unlike Prestrike B, an exponential increase in field emission current is observed before the prestrike. Although the field emission current in this case is lower in amplitude (under 10 mA) than in Prestrike A (which exceeds 10 mA), it still indicates a measurable contribution from field emission current. Upon prestrike initiation, the field emission current collapses to zero.

Field emission induced prestrike (FEPS) and particle-induced prestrike (PPS) can be distinguished by their characteristic field emission current and voltage waveforms. In FEPS, an exponential rise in field emission current is coupled with a corresponding exponential voltage decay, culminating in the simultaneous termination of both parameters at the prestrike moment. Prestrike A exhibits these features and is therefore classified as FEPS.

In contrast, PPS is characterized by the absence of significant field emission current prior to prestrike. It begins with a sudden drop in voltage, followed by partial insulation recovery. The reason for the sudden drop in voltage is the distortion of the electric field by the particles. As the contacts approach, the increasing electric field eventually exceeds the recovering dielectric strength, leading to secondary breakdown. This behavior is typified by Prestrike B, which is therefore identified as PPS.

Prestrike C exhibits a hybrid mechanism, featuring a measurable increase in field emission current before prestrike and a voltage rebound following initial breakdown. This distinctive combination classifies Prestrike C as a new prestrike type: Field Emission-Particle Induced Prestrike (FE-PPS).

### Statistical analysis of prestrike characteristics

**Fig 7:**
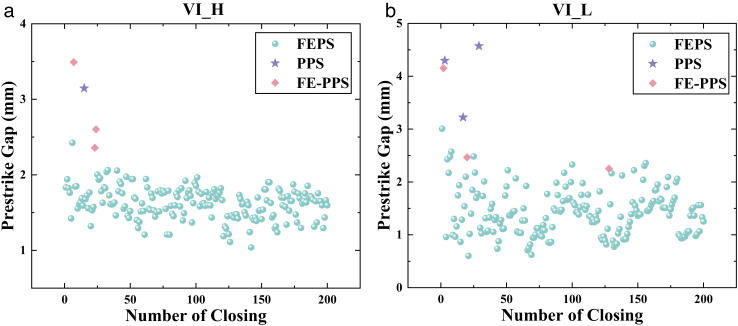
The experimental results of VI_H and VI_L for each closing process under DC voltage. (a). The experimental results of VI_H for each closing process under DC voltage. (b). The experimental results of VI_L for each closing process under DC voltage.

Fig 7 shows the experimental results of VI_H and VI_L closing 200 times under DC voltage. From Fig 7(a), it can be seen that among the types of prestrike occurring in VI_H, there are 196 times for FEPS, 1 time for PPS, and 3 times for FE-PPS. From Fig 7(b), it can be seen that among the types of prestrike occurring in VI_L, there are 194 times for FEPS, 3 times for PPS, and 3 times for FE-PPS. The prestrike types occurring in the VI are mainly FEPS, with PPS and FE-PPS occurring less frequently.

**Fig 8:**
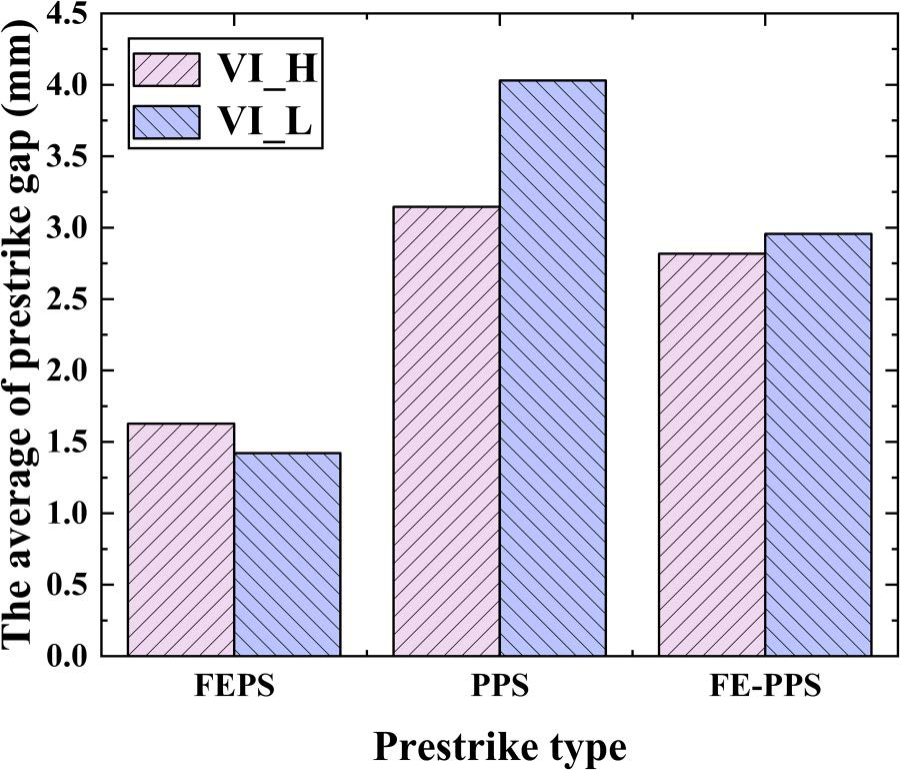
The average values of prestrike gap of the three prestrike types for VI_H and VI_L.

For VI_H, the prestrike gap ranges from 1.039 to 2.425 for FEPS, 3.1458 for PPS (one data only), and 2.359 mm to 3.492 mm for FE-PPS. For VI_L, the prestrike gap ranges from 0.601 mm to 3.007 mm for FEPS, 3.223 mm to 4.572 mm for PPS, and 2.250 mm to 4.153 mm for FE-PPS. Fig 8 illustrates average values of prestrike gap of the three prestrike types for VI_H and VI_L. For VI_H, the average value of prestrike gap is 1.628 mm for FEPS, 3.146 mm for PPS, and 2.817 mm for FE-PPS. For VI_L, the average value of prestrike gap is 1.422 mm for FEPS, 4.030 mm for PPS, and 2.956 mm for FE-PPS.

The cumulative probability distribution of the prestrike gap is an important parameter to investigate the prestrike characteristics. In this paper, the cumulative probability distribution of the VI under 30kV DC voltage level is obtained, which conforms to the two-parameter Weibull distribution, as shown in Equation (4):


$$F(d_{pre})=1-\exp\left[-{\left(\frac{d}{\psi }\right)}^{\lambda }\right]$$ (4)

Where: *ψ* is a shape parameter; *d*_*pre*_ is the distance between the contacts at prestrike moment; *λ* is the eigenvalue of *d*_*pre*_. The Weibull distribution shows the probability of prestrike when the gap length between two contacts is less than a given *d*_*pre*_*.*

**Fig 9:**
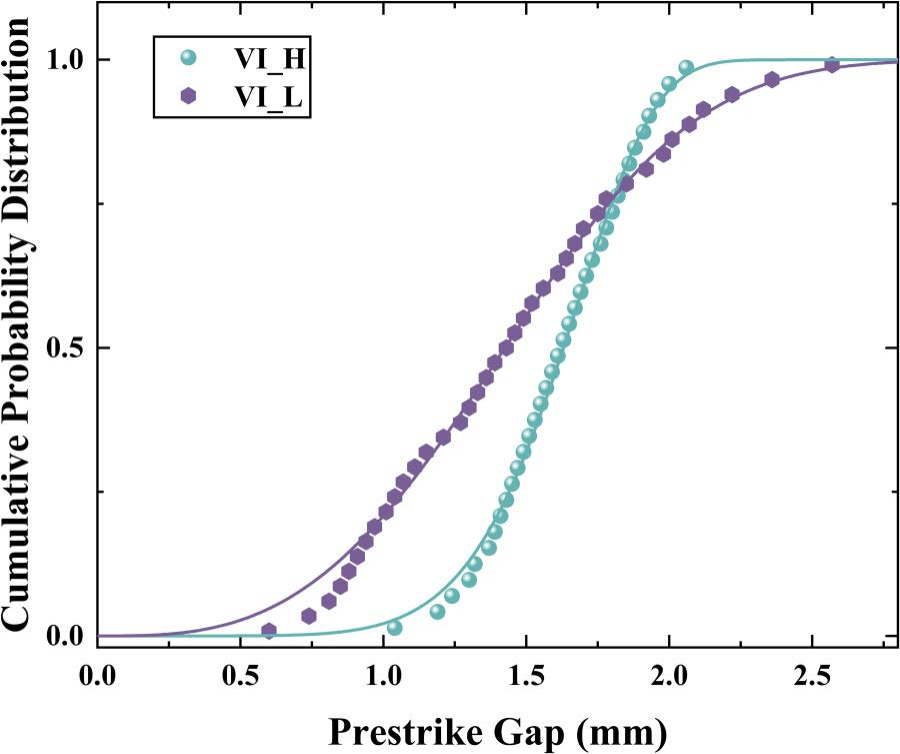
Cumulative probability distribution of prestrike gap for VI_H and VI_L.

*ψ* and *λ* were calculated from the experimental data as shown in Table 2. From the obtained *ψ* and *λ*, the fitting curves of the experimental data can be plotted. The characteristic parameters can be extracted from the distribution fitting curves, including the prestrike gap corresponding to 10% (*d*_*10*_), 50% (*d*_*50*_) and 90% (*d*_*90*_) cumulative probability. Taking *d*_*10*_ as an example, this parameter indicates that the probability of the prestrike will be increased to 10% when the distance between two contacts is less than this threshold [22]. The coefficient of determination *R*^*2*^ characterizes the explanatory power of the Weibull distribution for experimental datasets. Both VI_H and VI_L exhibit *R*^*2*^ values exceeding the threshold of 0.9, demonstrating that the distribution function effectively fits the experimental dataset. Fig 9 shows the cumulative probability distribution of the prestrike gap for the VI_H and VI_L.

**Table 2:** Parameter values of the Weibull distribution.

Experimental Group	*ψ*	*λ*	*R* ^*2*^
VI_H	1.7164	7.0828	0.9967
VI_L	1.6056	3.0711	0.9983

The dispersion of the prestrike gap is one of the key parameters for assessing the performance of controlled switching. This parameter *σ* is quantitatively characterized by calculating the standard deviation of the prestrike gap values, as shown in Equation (5):


$$\sigma =\sqrt{\frac{1}{N}{\sum {\mathrm{\backslash nolimits}}_{i=1}^{N}\left(d_{prei}-{\overset{―}{d}}_{pre}\right)}^{2}}$$ (5)

Where *N* is the number of each data set; ‾*d*_pre_ is the average value of prestrike gap for each data set; *d*_*prei*_ is the prestrike gap for the *i*_*th*_ closing operation. The dispersion of prestrike gap is shown in the third column of Table 3, which also lists the three quartiles of *d*_*10*_, *d*_*50*_ and *d*_*90*_. The above parameters are obtained based on the calculation of equation (4), which can systematically characterise the probability distribution of prestrike gap.

**Table 3:** The *d*_*10*_, *d*_*50*_ and *d*_*90*_ and the dispersion of prestrike gap under 30 kV DC voltage.

Experimental Group	DC Voltage/kV	*σ*/mm	*d*_*10*_/mm	*d*_*50*_/mm	*d*_*90*_/mm
VI_H	30	0.278	1.931	1.630	1.249
VI_L	30	0.5787	2.108	1.425	0.773

**Fig 10:**
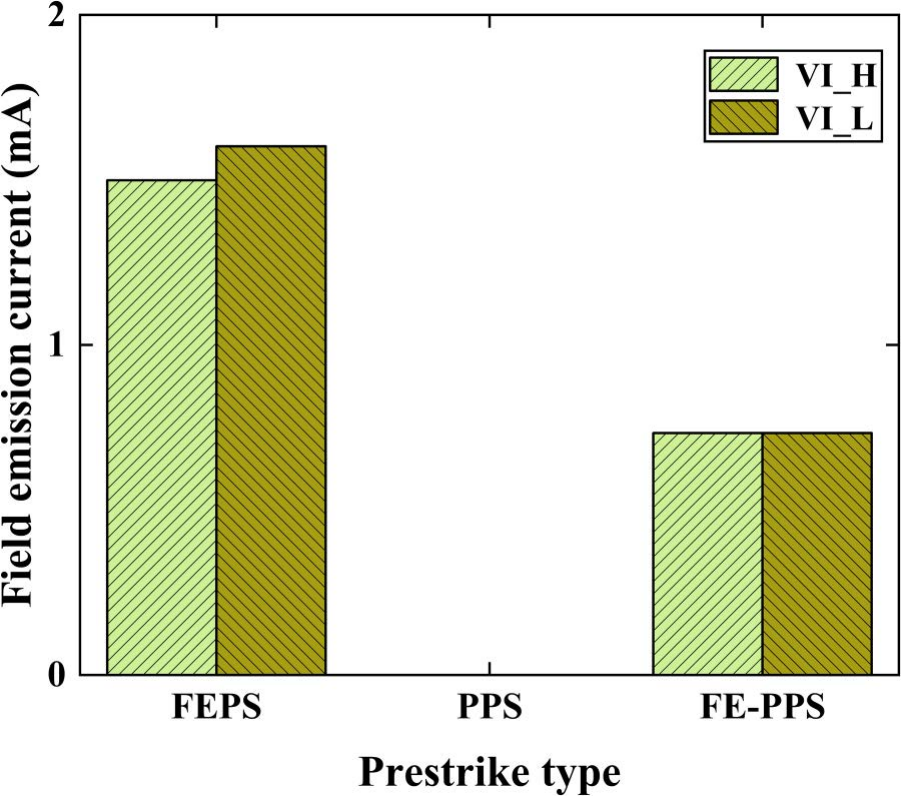
Average values of field emission current of the three prestrike types for VI_H and VI_L at prestrike moment.

Fig 10 illustrates average values of field emission current of the three prestrike types for VI_H and VI_L at prestrike moment. For VI_H, the average value of field emission current at prestrike moment is 1.499 mA for FEPS, 0 mA for PPS, and 0.733 mA for FE-PPS. For VI_L, the average value of field emission current at prestrike moment is 1.602 mA for FEPS, 0 mA for PPS, and 0.733 mA for FE-PPS.

## Discussion

A summary of the three prestrike types in VI_H and VI_L shows: FEPS occurred 390 times, FE-PPS occurred 6 times, and PPS occurred 4 times. FEPS occurred most frequently, accounting for 97.5% of the total. FE-PPS had a slightly higher frequency than PPS, accounting for 1.5%. PPS was the least frequent, accounting for 1%. The above results reflect the difference in the likelihood of occurrence among the three types of prestrike. The following discussion combines the physical explanations of these three prestrike types to analyze this result.

**Fig 11:**
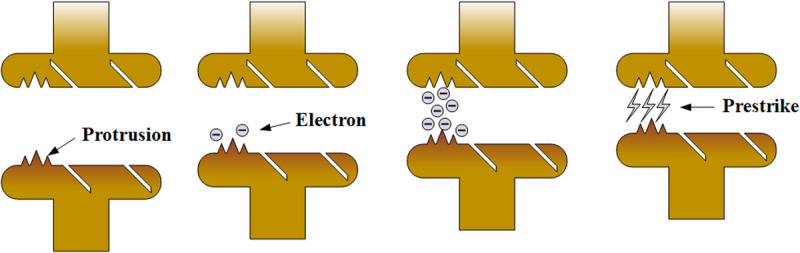
Schematic diagram of the physical explanation of the FEPS.

Fig 11 illustrates the physical mechanism underlying FEPS. During capacitor banks switching, the inrush current generates localized fusion welds on the contact surface. Upon subsequent separation, these welds fracture, forming surface defects such as micro-protrusions, pits, and metallic particles. When DC voltage is reapplied, these defects intensify the local electric field, resulting in high-density field emission current. The enhancement of the electric field strength leads to an increase in the field emission current. These currents induce localized heating through Joule and Nottingham effects [23], leading to thermal instability under the combined influence of elevated current density, strong electric fields, and potential ion bombardment. Field emission current reaching a certain threshold can cause explosive electron emission [24] or thermal runaway process [25], ultimately resulting in prestrike. Based on the above analysis, the occurrence of FEPS is related to the electric field strength. When the voltage remains constant, the electric field strength depends on the distance between the contacts. FEPS has the least strict occurrence conditions, hence it appears most frequently.

**Fig 12:**
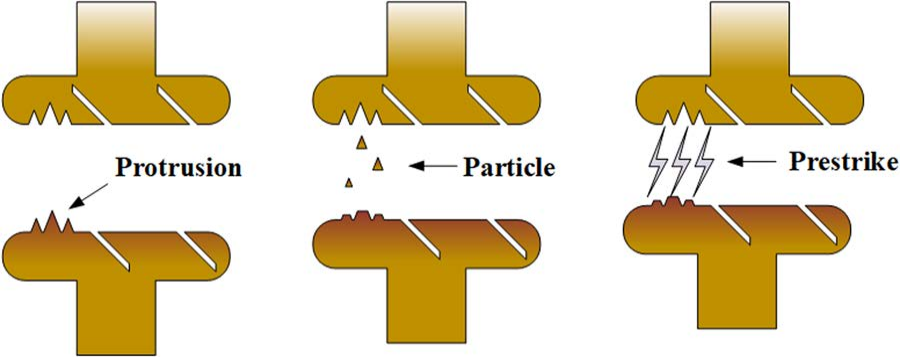
Schematic diagram of the physical explanation of the PPS.

Fig 12 depicts the physical mechanism of PPS. The origin of metallic particles on contact surfaces can be broadly categorized into three sources: (1) residual particles from electrode manufacturing or environmental dust embedded via Van der Waals forces [26]; (2) debris generated by mechanical abrasion [27] and arc erosion during switching operations [28,29]; (3) thermally induced detachment resulting from localized field emission heating and associated instabilities [30]. In the present study, non-metallic particles were found to have negligible impact on breakdown voltage [31]; Therefore, the particles discussed in this paper can be considered metal particles. The VI contacts in this study used CuCr30 material. Thus, the particles discussed in this paper are primarily composed of CuCr30.

Under the influence of the electric field, these particles will be polarized and detach from the contact surface. Mechanical vibrations can further promote particle detachment [32]. If the particles fall within a critical size and the electric field strength is sufficient, they gain kinetic energy while accelerating across the gap. Upon impact with the opposing electrode, their kinetic energy is converted into heat, causing localized melting and vaporization. The resulting metal vapor undergoes collisional ionization with field-emitted electrons, thereby initiating prestrike [33,34]. Based on the above analysis, the occurrence of PPS depends only on particles. PPS has the strictest occurrence conditions, requiring particles to enter the vacuum before field emission current develops. Therefore, PPS appears least frequently.

**Fig 13:**
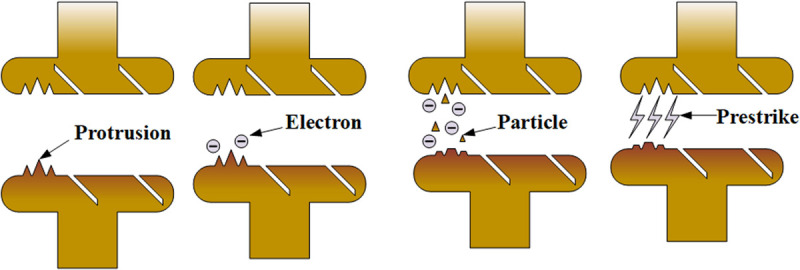
Schematic diagram of the physical explanation of the FE-PPS.

Fig 13 illustrates the physical mechanism of FE-PPS. As the contact gap narrows during closing, the electric field strength increases. Micro-defects on the contact surface induce field emission current. Unlike PPS, the generation of particles in FE-PPS is directly related to field emission current. This current cause localized heating at micro-protrusions. The combined effect of thermomechanical stress and intensified local field facilitate the detachment of particles. Once the particles are released from the contact, they distort the local electric field and cause prestrike to occur. Based on the above analysis, FE-PPS occurs due to particles created by the heating effect of field emission current. Local heating from this current causes particles to detach from contacts, enter the vacuum, and ultimately trigger prestrike. Generating particles for FE-PPS is slightly easier than for PPS. This means FE-PPS has less demanding occurrence conditions than PPS. Consequently, FE-PPS appears more frequently than PPS.

**Fig 14:**
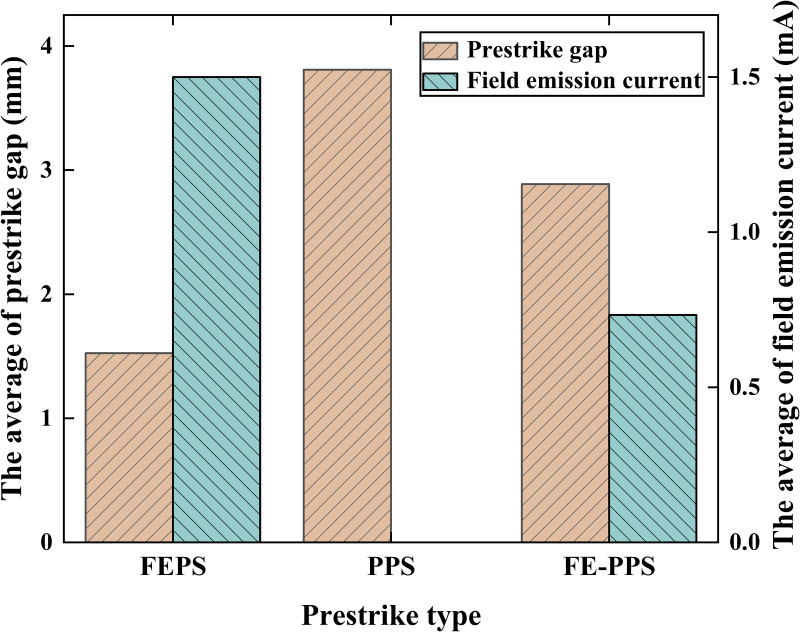
The average of the prestrike gap and the corresponding field emission current at prestrike moment for the three prestrike types.

Fig 14 presents the average of the prestrike gap and the corresponding field emission current at prestrike moment for the three prestrike types. The average of prestrike gap for FEPS, PPS, FE-PPS are 1.525 mm, 3.809 mm and 2.887 mm respectively. FEPS exhibits the smallest average prestrike gap, PPS is the largest, and FE-PPS falls in between. In FEPS, the decreasing contact distance enhances the vacuum gap’s electric field, which reaches a critical value required to initiate prestrike, resulting in a minimal prestrike gap. In PPS, particle motion-driven by electrostatic forces and mechanical vibration-can cause breakdown even at relatively wide contact gaps, leading to the largest prestrike gap. In FE-PPS, particle detachment is induced by field emission heating; prestrike occurs only after the current sufficiently heats the erosion area and emit particles. Hence, the prestrike gap in FE-PPS is intermediate.

Additionally, the average values of field emission currents at prestrike moment for FEPS, PPS, and FE-PPS are 1.5 mA, 0 mA and 0.733 mA, respectively. The field emission current of FE-PPS at the prestrike moment is measured to be 48.89% of that exhibited by FEPS. This finding confirms the interpretation proposed in this study concerning FE-PPS: the heating effect induced by field emission current causes particle detachment from contact surface, thereby triggering the prestrike phenomenon.

**Fig 15:**
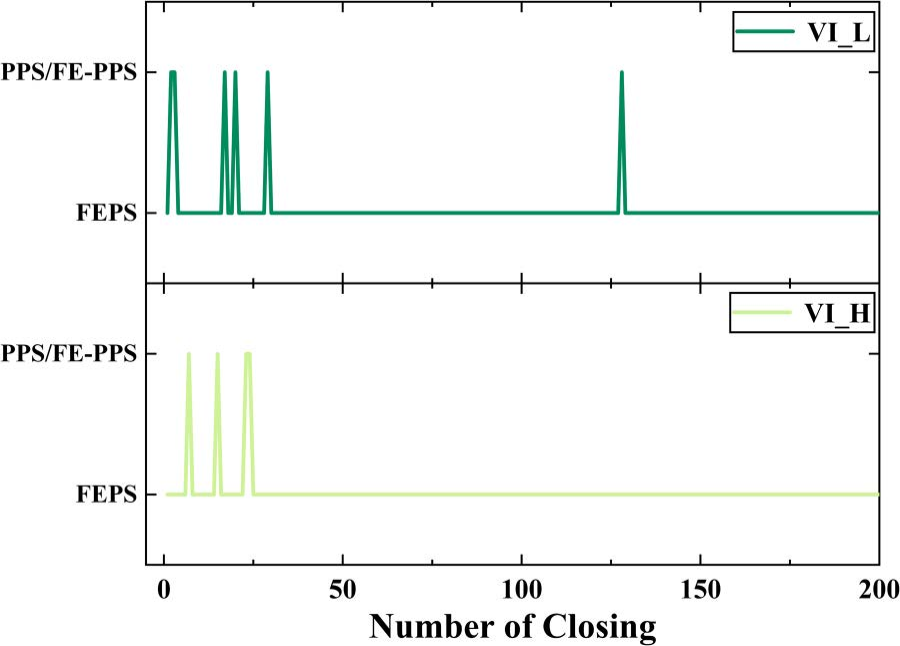
Statistical results of the type of prestrike.

As shown in Fig 15, prestrike events involving particles (PPS and FE-PPS) predominantly occur within the first 50 closing operations, with only one instance recorded beyond this point. This trend is consistent for both VI_H and VI_L. The experimental procedure first involves capacitive switching experiment. Subsequent closing operations under DC voltage are then performed 200 times. Capacitive switching induces surface defects on the contacts. Thus, the initial 50 closing operations represent the most severe phase of contact surface degradation. As the number of closings increases, some surface irregularities are mechanically worn down, improving surface conditions and reducing the occurrence of PPS and FE-PPS.

It should be noted that the experimental findings are not limited to the 30 kV DC voltage. In reactive power compensation applications, when VI contacts close capacitor banks, inrush current erosion induces surface defects on the contacts. These defects degrade the internal insulation of the VI, altering prestrike characteristics and affecting prestrike types.

At higher DC voltage, field emission current during VI closing increases significantly. This enhances the thermal effects of field emission current, increasing the occurrence frequency of FE-PPS. Conversely, reducing DC voltages will decrease field emission current. Under such condition, particle generation primarily relies on contact collisions, leading to a higher PPS occurrence frequency. This analysis indicates that during AC voltage operations, dynamically changing voltages during VI closing introduce randomness in the occurrence frequencies of different prestrike types.

## Conclusion

This study investigates prestrike phenomena in VI eroded by inrush current (Amplitude: 20 kA, Frequency: 4250 Hz). Based on experimental observations, three distinct types of prestrike events occurring during the closing process are identified and analyzed. The main conclusions are summarized as follows:

1. Three types of prestrike (FEPS, PPS, and FE-PPS) are identified in eroded VIs under DC voltage. For FEPS, the enhancement of the electric field strength leads to an increase in the field emission current, and the field emission current reaches a certain threshold that leads to the occurrence of prestrike. For PPS, particles cause distortion of the electric field in the vacuum gap, which in turn causes the occurrence of prestrike. For FE-PPS, the heating of the field emission current leads to the generation of particles, which distort the electric field and cause the occurrence of prestrike.
2. Correlation of prestrike type with prestrike gap and field emission current is explored. The average of prestrike gap for FEPS, PPS, FE-PPS were 1.525 mm, 3.809 mm and 2.887 mm respectively. PPS occurs without an accompanying field emission current. The FE-PPS occurs with a field emission current that is about 48.89% of the field emission current of the FEPS occurrence.
3. The prestrike characteristics of VI after inrush current erosion were obtained. For VI_H, its prestrike gaps of *d*_*10*_, *d*_*50*_ and *d*_*90*_ were 1.931 mm, 1.630 mm and 1.249 mm, respectively. For VI_L, its prestrike gaps of *d*_*10*_, *d*_*50*_ and *d*_*90*_ were 2.108 mm, 1.425 mm and 0.773 mm, respectively. The dispersions of prestrike for VI_H and VI_L were 0.278 mm and 0.579 mm, respectively. These results have important implications for the development of closing strategies for VCB controlled switching.

In the next step of the study, a VCB controlled switching strategy considering the effect of inrush current erosion will be developed based on the prestrike characteristics of VI after inrush current erosion. On the other hand, the condition of prestrike during the closing process may reflect the degree of erosion of the contact surface. Combined with the experimental datas of prestrike, a diagnostic method for the deterioration degree of VI contact surface will be proposed.

## Supporting information

S1 Prestrike data. (XLSX)
